# Altered liver expression of genes involved in lipid and glucose metabolism in mice with partial IGF-1 deficiency: an experimental approach to metabolic syndrome

**DOI:** 10.1186/s12967-015-0684-9

**Published:** 2015-10-14

**Authors:** J. Rodríguez De Ita, I. Castilla-Cortázar, G. A. Aguirre, C. Sánchez-Yago, M. Olleros Santos-Ruiz, L. Guerra-Menéndez, I. Martín-Estal, M. García-Magariño, V. J. Lara-Díaz, J. E. Puche, U. Muñoz

**Affiliations:** Escuela de Medicina, Tecnologico de Monterrey, Avenida Morones Prieto No. 3000 Pte. Col. Los Doctores, 64710 Monterrey, Nuevo León México; Fundación de Investigación HM Hospitales, Plaza. del Conde del Valle de Súchil 16, 28015 Madrid, Spain; Department of Medical Physiology, Institute of Applied Molecular Medicine (IMMA), School of Medicine, University CEU San Pablo, Calle Boadilla del Monte s/n, km 5,3, 28668 Madrid, Spain

**Keywords:** Metabolism, Metabolic syndrome, IGF-1, Type 2 diabetes, Growth hormone, Gluconeogenesis, Glucogenolysis, Glucose-6-phosphate (G6P), Phosphoenolpyruvate carboxykinase (PEPCK), ATP-citrate lyase (*Acly*), Acetyl-CoA acyltransferase (*Acaa1b*), Insulin resistance, Oxidative damage, Dyslipidemia

## Abstract

**Background:**

Insulin growth factor 1 (IGF-1) has multiple effects on metabolism. Much evidence suggests that the deficiency of this hormone increases insulin resistance, impairs lipid metabolism, augments oxidative damage and deregulates the neuro-hormonal axis. An inverse relationship between IGF-1 levels and the prevalence of Metabolic Syndrome (MetS) with its cardiovascular complications has been identified. However, the underlying mechanisms linking IGF-1 and MetS are still poorly understood. In order to elucidate such mechanisms, the aim of this work was to study, in mice with partial IGF-1 deficiency, liver expression of genes involved in glucose and lipid metabolism as well as serum levels of glucose, triglycerides and cholesterol, as well as liver malondialdehyde (MDA) levels, as a marker for oxidative damage.

**Methods:**

Three experimental groups were studied in parallel: Controls (CO), wild type mice (*igf*-*1*^+/+^); untreated heterozygous mice (Hz, *igf*-*1*^+/−^) and Hz (*igf*-*1*^+/−^) mice treated with low doses of IGF-1 for 10 days (Hz + IGF-1).

**Results:**

A reduction of IGF-1 serum levels in the Hz group was found, which was normalized by IGF-1 therapy. Serum levels of glucose, triglycerides and cholesterol were significantly increased in the untreated Hz group as compared to both controls and Hz + IGF-1 groups. The expression of genes involved in gluconeogenesis, glycogenolysis, lipid synthesis and transport, and catabolism were altered in untreated Hz animals and the expression of most of them was normalized by IGF-1 therapy; MDA was also significantly increased in the Hz untreated group.

**Conclusions:**

The mere partial IGF-1 deficiency is responsible for the reduction in the expression of genes involved in glucose and lipid metabolism, resulting in dyslipidemia and hyperglycemia. Such genetic alterations may seriously contribute to the establishment of MetS.

## Background

Insulin-like growth factor 1 (IGF-1) is an anabolic hormone mainly produced in the liver by growth hormone (GH) endocrine stimulus [1]. IGF-1 possesses multiple effects on metabolism [2].

Interestingly, GH and insulin act in symphony with IGF-1 to produce a harmonious and coordinated response [3–5]. There is a yearly increasing number of studies suggesting the role of IGF-1 in metabolic coordination [2]. Accumulated evidence has proven an IGF-1 implication in lipid and glucose metabolism [5, 6].

Recent data support that IGF-1 deficiency increases insulin resistance, impairs lipid metabolism, promotes oxidative damage and deregulates the neuro-hormonal axis [7–9]. IGF-1 circulating levels decrease with aging, and such a decrease is associated with insulin resistance and dyslipidemia. Interestingly, IGF-1 replacement therapy improved the overall homeostasis [7]. On the other hand, an inverse relationship between IGF-1 circulating levels and incidence of metabolic syndrome (MetS) with liver steatosis, insulin resistance, hyperlipidemia and visceral obesity has been identified [10–13]. In addition, cardiovascular complications of MetS have been also reported [14].The majority of the studies have found that patients with MetS suffer more from cardiovascular disease (CVD) and an increased predisposition towards developing it [15–18].

Moreover, the MetS is also a good predictor for the development of type 2 Diabetes (T2D) [19–21]. Insulin resistance, hyperinsulinemia, dyslipidemia and obesity precede the progression to T2D [22] and the presence of MetS increases up to five fold the risk for T2D as compared to controls [21, 22]. The risk is increased up to six- to sevenfold, if insulin resistance is also present [23].

On the other hand, MetS is strongly related with insulin resistance and obesity, as well as non-alcoholic fatty liver disease (NAFLD), polycystic ovarian syndrome, hypogonadism and microvascular disease among others [23–25].

In this physiopathological context the underlying mechanism between the IGF-1 deficiency and the establishment of MetS is poorly understood. In order to gain more insight into these mechanisms, an experimental model of partial IGF-1 deficiency was used [26]. The experimental protocol included three groups of adult mice (28 ± 6 weeks old): untreated, heterozygotes (igf1^+/−^) mice with partial IGF-1 deficiency; heterozygotes (igf1^+/−^) mice treated with IGF-1; and wild type (igf1^+/+^) mice that served as controls.

The specific aim of this work was to investigate whether the mere IGF-1 deficiency is able to alter the expression of genes involved in glucose and lipid metabolic pathways. Thus, liver gene expression studies carried out by microarray technique followed by RT-qPCR confirmation; serum levels of IGF-1, glucose, cholesterol and triglycerides were determined as well as MDA in liver homogenates, all studied in the three experimental groups.

## Methods

### Animals and experimental design

The experimental model was established and characterized as previously reported [26]. Briefly, IGF-1 heterozygous mice were obtained by cross-breeding transgenic mice, line 129SV and Igf1^tm1Arge^ [27].

Animal genotype determination was performed by PCR analysis (Applied Biosystems, 2720 Thermal Cycler, Spain). DNA was extracted from a piece of tail and specific primers were used to identify both *igf*-*1* and *neo* genes (Extract-N-Amp TM Tissue PCR KIT Sigma, USA).

Animals were housed in cages in a room with a 12-h light/dark cycle, constant humidity (50–55 %) and temperature (20–22 °C). Food (Teklad Global 18 % protein rodent diet, Harlan Laboratories, Spain) and water were given ad libitum. All experimental procedures were performed in compliance with The Guiding Principles for Research Involving Animals and approved by the Bioethical Committee from our institution.

Three groups of male mice 28 ± 6 weeks old were included in the experimental protocol: Controls wild type mice (CO, *igf*-*1*^+/+^, n = 10); untreated heterozygotes mice (Hz, *igf*-*1*^+/−^, n = 10) and heterozygous animals subcutaneously treated with IGF-1 (2 µg/100 g body weight/day) for 10 days (Hz + IGF-1, *igf*-*1*^+/−^, n = 10). Both the CO and Hz groups received vehicle (succinate buffer). IGF-1 was provided by Chiron Corporation, USA.

On the 11th day mice were weighed, blood was obtained from submandibular vein and thereafter animals were sacrificed by cervical dislocation. The liver was carefully dissected out, weighed (Denver Instrument, Germany) and divided in 2 sections: left lobe was stored in RNAlater (Qiagen-Izasa, Spain) at −80 °C for microarray and PCR RNA analyses, and right lobe for histology and MDA assessment.

### Serum and liver analysis

Serum levels of IGF-1 were determined by ELISA in a Varioskan spectrophotometer (Thermo Scientific, Finland), following specific commercial assay protocol instructions (Chiron Corporation, USA).

The serum concentrations of glucose, triglycerides and cholesterol were determined by routine laboratory methods by a COBAS INTEGRA 400 Plus auto analyzer (Roche-Hitachi, Germany), Calibration Reagents (Roche) and Cassettes of the same brand.

### Malondialdehyde levels

Malondialdehyde (MDA) was used as an index of lipid peroxidation and was measured after heating samples at 45 °C for 60 min in acid medium. It was quantitated by a colorimetric assay using LPO-586 (Bioxytech; OXIS International Inc., Portland, OR, USA), which, after reacting with MDA, generates a stable chromophore that can be measured at 586 nm (Hitachi U2000 Spectro; Boehringer Mannheim). Determinations were performed in homogenates of liver tissue in Tris–HCl solution (1 g of liver tissue per 10 ml) centrifuged at 3000*g* during 10 min at 4 °C.

### Gene expression studies

#### Microarray analysis

Liver mRNA was isolated from animals belonging to the three experimental groups in accordance with the protocol outlined in RNAqueousH-Micro Kit (Ambion, USA). Technical procedures for microarray analysis, including quality control of mRNA, labeling, hybridization and scanning of the arrays were performed according to standard operating procedures for Affymetrix protocols (GeneChipH Expression Analysis Manual, Affymetrix, USA). The mRNAs were profiled using Affymetrix HT MG-430. The array signals were normalized using Robust Multichip Averages [28] and batch-effects of the three replicates were corrected using ComBat [29]. Differentially expressed genes between Hz + IGF-1 and CO samples were selected using FDR-corrected *p* value of 0.01 (p value of < 0.05).

#### Total RNA extraction, reverse transcription and quantitative real time polymerase chain reactions (RT-qPCR)

Hepatic lobules were cryopreserved in RNAlater (Qiagen-Izasa, Spain). The day performing PCR determinations hepatic samples were homogenized with TRIzol reagent (Invitrogen, UK) by Tissue Lyser LT (Qiagen-Izasa, Spain) and RNA was extracted and purified using the (Qiagen), RNeasy Mini Kit including digestion with RNase-free DNase, following the manufacturer’s instructions. RNA quality was checked by the A260:A280 ratio and with Bioanalyzer 2100 (Agilent Technologies Inc., USA). Purified RNA was then converted to cDNA by using the RNA-to-DNA EcoDryTM Premix (Clonetech Labs, USA) for qPCR assays. Quantitative real time PCR assays were performed in a 3100 Avant Genetic Analyzer (Applied Biosystems Hispania, Spain). The thermal profile consisted of an initial 5 min melting step at 95 °C followed by 40 cycles at 95 °C for 10 s and 60 °C for 60 s.

Specific Taqman^®^ probes for the selected genes (*Acaa1b*, *Acat1*, *Acsl1*, *Acot9*, *Fabp1*, *Fabp5*, *Hmgcs1*, *Lpl*, *Lrp1*, *Pcsk9*, *Ankra2*, *Hmgcr*, *G6pc*, *Pck1*, *Pdk4*, *Acly*, *Igf1*, *Igfbp1*, and *Igfbp3*) were supplied by Applied Biosytems.

The relative mRNA levels of the genes of interest were normalized to Tbp expression using the simplified comparative threshold cycle delta, cycle threshold (CT) method [2^−(ΔCT gene of interest − ΔCT actin)^] [30]. Tbp was selected fot endogenous control after carefully analyzing 12 genes (Actb, B2m, Gapdh, Gusb, Hsp90ab1, Ldha, Pgk1, Ppih, Sdha, Tbp, Tfrc, and Ubc), which are widely used as housekeeping genes. Only 5 out of these 12 did not vary their expression when comparing Hz with Controls. We assayed those 5 genes and selected the one, which showed more stable and reproducible values, which was Tbp, a very stable gene coding for a transcription factor that binds the TATA box.

### Statistical analysis

All data represent mean ± SEM. Statistical analysis was performed on SPSS 20 (Statistical Package for Social Sciences, USA). Significance was estimated by the Kruskal–Wallis ANOVA followed by a post hoc test for distribution-free multiple comparisons (Bonferroni). Correlation between IGF-1 and weight was analyzed by Spearman test. Differences were considered significant at a level of p < 0.05.

## Results

### IGF-1 circulating levels, body and liver weight

**Fig. 1:**
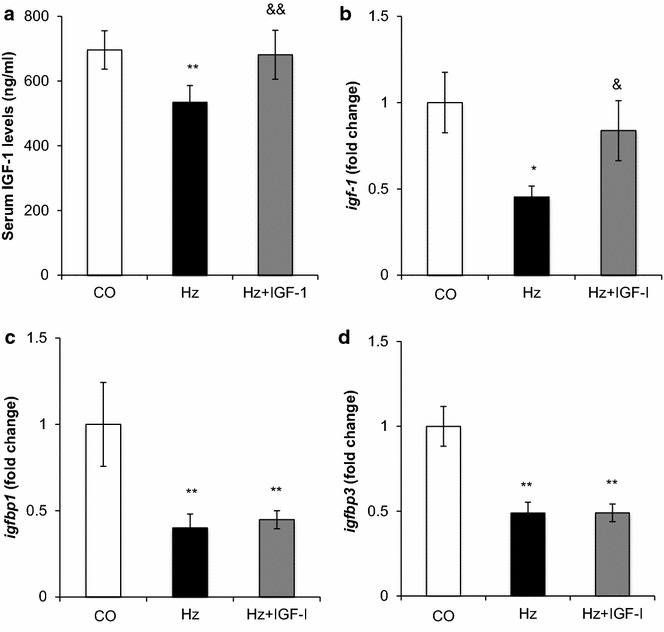
IGF-1 serum levels. IGF-1 and IGFBPs liver gene expression.** a** IGF-1 circulating levels at the end of the treatment determined in the three experimental groups.** b** Liver expression of *igf-1*,** b** igfbp1 and,** d** *igfbp3* genes in the three experimental groups determined by RT-qPCR and expressed as relative values compared to controls (fold change). **p < 0.01,*p < *0.05 vs. CO;* ^&&^ *p < 0.01*, ^&^ *p <* *0.05 vs. Hz*

According to previous series [26, 31] 28 ± 6 weeks old Hz mice showed a significant decrease of circulating levels of IGF-1 as compared to CO. The exogenous administration of low doses of IGF-1 normalized IGF-1 serum levels in Hz + IGF-1. Thus, the usefulness of the substitutive IGF-1 therapy at low doses was confirmed (see Fig. 1a).

As an anabolic hormone, IGF-1 deficiency induced body weight loss in untreated Hz mice as compared to controls.

Interestingly, IGF-1 treatment for only 10 days was able to restore the bodyweight (CO: 39.76 ± 2.45 g vs. Hz 33.59 ± 1.06 g, p < 0.01; Hz + IGF-1: 39.52 ± 1.48 g vs. Hz p < 0.01). Absolute liver weight was significantly decreased in Hz mice as compared to CO (Hz: 1.63 ± 0.09 g vs. CO: 1.92 ± 0.16 g, p < 0.05), while differences between CO and Hz + IGF-1 mice were not found (Hz + IGF-1: 1.93 ± 0.19 g, p = ns).

### Serum and liver analysis

Table 1 summarizes serum glucose, triglycerides and cholesterol values and hepatic levels of MDA, as a marker of oxidative damage in the three experimental groups. Untreated Hz mice showed a significant increase in all these parameters. IGF-1 replacement therapy had the property to reduce, although not to completely abolish, the increases in glucose, triglycerides and cholesterol. Furthermore, it was able to diminish hepatic MDA to similar levels to those found in controls.

**Table 1:** Serum and liver parameters for the three experimental groups

	Controls	Untreated IGF-1 deficient mice	Treated IGF-1 deficient mice
Glucose (mg/dL)	83.07 ± 5.62	*123.91* *±* 11.27**	93.59 ± 3.95^&^
Triglycerides (mg/dL)	235.70 ± 11.50	*291.01* *±* 21.50*	254.11 ± 12.20^&^
Cholesterol (mg/dL)	221.76 ± 8.27	*271.43* *±* 12.76*	235.28 ± 4.86^&^
MDA (UM/mg protein/mL)	0.12 ± 0.02	*0.16* *±* 0.02*	0.07 ± 0.004^&^

Control group, WT mice; Hz group including untreated mice with partial IGF-1 deficiency; Hz + IGF-1 group: Hz mice treated with low doses of IGF-1 during 10 days

** p < 0.01, * p < 0.05 vs. Controls; ^&^ p < 0.05 vs. Hz group

### Liver gene expression studies

Microarray technique revealed several genes (Table 2) either hypo- or hyper-expressed in Hz as compared to CO mice (fold change over ±1.5), as well as Hz + IGF-1 animals compared to the Hz group.

**Table 2:** Liver expression of genes related to IGF-1

Protein	Gene	Hz vs. WT (fold change)	p value	Hz + IGF vs. Hz (fold change)	p value
Insulin-like growth factor 1	*(Igf1)*	−2.48	0.0017	1.64	0.002
Insulin-like growth factor 2	*(Igf2)*	−1.35	0.14	1.48	0.16
Insulin-like growth factor binding protein 1	*(Igfbp1)*	−4.53	0.0001	−2.2	0.0012
Insulin-like growth factor binding protein 2	*(Igfbp2)*	−1.18	0.015	−1.02	0.37
Insulin-like growth factor binding protein 3	*(Igfbp3)*	−1.78	0.012	1.33	0.011
Insulin-like growth factor binding protein 4	*(Igfbp4)*	1.13	0.33	1.35	0.29
Insulin-like growth factor binding protein 5	*(Igfbp5)*	−1.37	0.10	1.37	0.17
Insulin-like growth factor binding protein 6	*(Igfbp6)*	−1.10	0.31	1.19	0.12
Albumin	*(Alb)*	−1.06	0.12	−1.01	0.14
Parvalbumin	*(Pvalb)*	−1.04	0.11	−1.10	0.13
Lactalbumin, alpha	*(Lalba)*	−1.10	0.14	1.04	0.12
D site albumin promoter binding protein	*(Dbp)*	2.33	0.05	−1.16	0.19

Control group, WT mice; Hz group including untreated mice with partial IGF-1 deficiency; Hz + IGF-1 group: Hz mice treated with low doses of IGF-1 during 10 days. Underlined values correspond to those with a fold-change >1.5, considered as a significant variation in the gene expression

### IGF-1 and main IGFBPs gene expression

Among the genes with an altered expression, we firstly focused on those closely related to the physiology of IGF-1 (Table 2). Real time quantitative PCR was performed to confirm changes over ±1.5-fold variance, such as IGF-1 and IGFBP-1 and IGFBP-3 (Fig. 1b–d).

Statistical differences were confirmed by RT-qPCR for *igf*-*1*, *igfbp1* and *igfbp3* genes. In the Hz group, *igf*-*1* expression was found significantly reduced and, unexpectedly, IGF-1 replacement therapy increased hepatic *igf*-*1* expression (Fig. 1b).

On the other hand*, igfbp1* and *igfbp3* expressions were found significantly reduced in untreated Hz animals and substitutive IGF-1 treatment did not induce any change on the expression of these two genes (Fig. 1c, d).

### Expression of genes involved in glucose metabolism

**Fig. 2:**
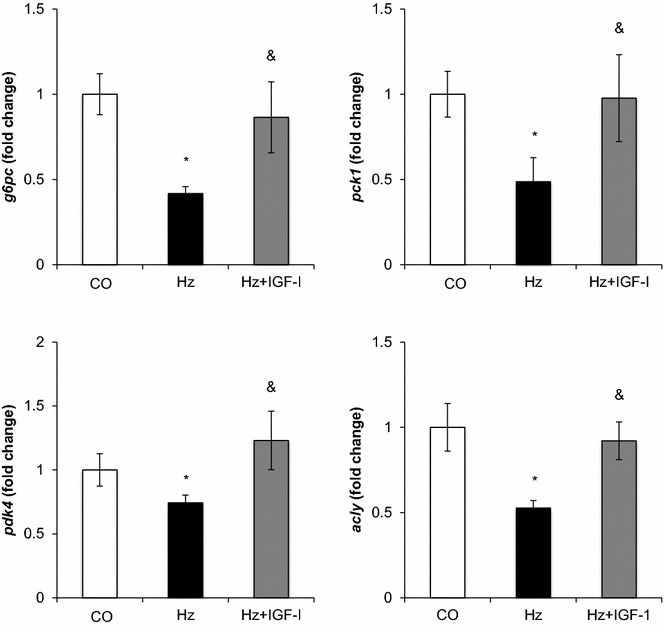
Liver expression of genes involved on gluconeogenesis and glycogenolysis. Expression of *g6pc* (Glucose-6-phosphatase), *pck1* (Phosphoenolpyruvate-carboxylase 1)*, pdk4* (Pyruvate dehydrogenase kinase isoenzyme 4) and *acly* (ATP-citrate lyase) genes determined by RT-qPCR and expressed as relative values compared to controls (fold change). *p < *0.05 vs. CO; * ^&^ *p*< *0.05 vs. Hz*

The expression of genes involved in glucose metabolism was analyzed by microarray analysis. RT-q PCR was performed to confirm changes over ±1.5 fold change (Table 3). Gene expression of *g6pc* (glucose-6-phosphatase, catalytic), *pck1* (phosphoenolpyruvate carboxykinase 1, cytosolic), pdk4 (pyruvatedehydrogenase kinase, isoenzyme 4) and *acly* (ATP citratelyase) was found significantly diminished in Hz as compared to CO. Interestingly, low doses of IGF-1 were able to normalize all these values to those found in the CO group (Fig. 2).

**Table 3:** Liver expression of genes related to glucose metabolism

Protein	Gene	Hz vs. WT (fold change)	p value	Hz + IGF vs. Hz (fold change)	p value
Fructosebisphosphatase 1	*(Fbp1)*	1.40	0.02	−1.49	0.01
Fructosebisphosphatase 2	*(Fbp2)*	1.64	0.017	1.06	0.42
*Glucose*-*6*-*phosphatase, catalytic*	*(G6pc)*	−1.92	0.001	−1.71	0.0059
Glucose 6 phosphatase, catalytic, 3	*(G6pc3)*	−1.01	0.56	1.31	0.017
*Phosphoenolpyruvatecarboxykinase 1, cytosolic*	*(Pck1)*	−2.42	0.001	1.35	0.08
Phosphoenolpyruvatecarboxykinase 2 (mitochondrial)	*(Pck2)*	−1.14	0.45	1.10	0.40
Pyruvate carboxylase	*(Pcx)*	−1.01	0.18	1.05	0.09
Pyruvate dehydrogenase kinase, isoenzyme 1	*(Pdk1)*	−1.46	0.0035	1.23	0.19
Pyruvate dehydrogenase kinase, isoenzyme 2	*(Pdk2)*	−1.17	0.03	1.33	0.035
Pyruvate dehydrogenase kinase, isoenzyme 3	*(Pdk3)*	1.23	0.42	−1.01	0.94
*Pyruvate dehydrogenase kinase, isoenzyme 4*	*(Pdk4)*	−1.83	0.03	−1.01	0.85
Pyruvate dehyrogenase phosphatase catalytic subunit 2	*(Pdp2)*	−1.08	0.09	1.21	0.04
*ATP citratelyase*	*(Acly)*	−1.67	0.0007	1.60	0.006
Aconitase 1	*(Aco1)*	−1.30	0.38	1.17	0.23
Aconitase 2, mitochondrial	*(Aco2)*	−1.18	0.013	1.16	0.85
Dihydrolipoamide S-acetyltransferase	*(Dlat)*	1.03	0.13	−1.15	0.19
Dihydrolipoamidedehydrogenase	*(Dld)*	−1.02	0.03	1.11	0.73
Dihydrolipoamide S-succinyltransferase	*(Dlst)*	1.14	0.40	1.41	0.01
Isocitratedehydrogenase 1 (NADP+), soluble	*(Idh1)*	1.07	0.04	−1.38	0.07
Isocitratedehydrogenase 2 (NADP+), mitochondrial	*(Idh2)*	−1.10	0.45	1.01	0.25
Isocitratedehydrogenase 3 (NAD+) alpha	*(Idh3a)*	−1.21	0.09	1.06	0.01

Control group, WT mice; Hz group including untreated mice with partial IGF-1 deficiency; Hz + IGF-1 group: Hz mice treated with low doses of IGF-1 during 10 days. Underlined values correspond to those with a fold-change >1.5, considered as a significant variation in the gene expression

### Lipid metabolism gene expression

**Fig. 3:**
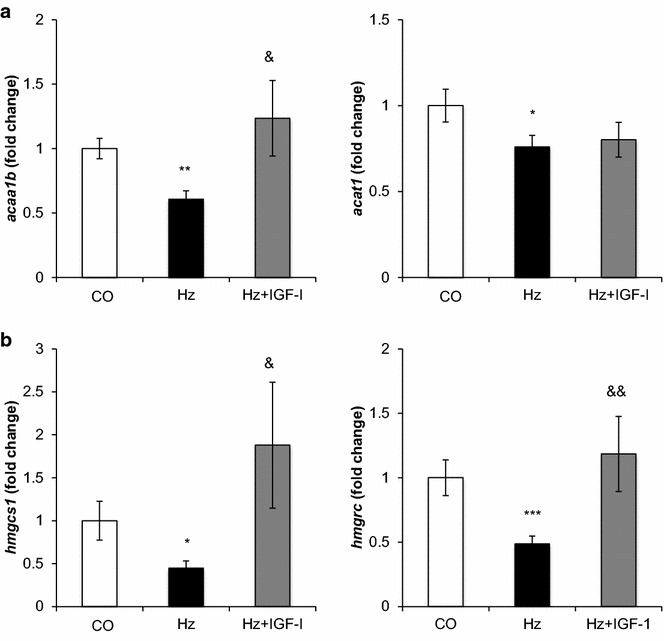
Liver expression of genes implicated in lipid metabolism.** a** Gene expression of *acaa 1b* (Acetyl-CoA acyltransferase 1B) and *acat1* (Acetyl-CoA acetyltransferase 1), implicated in lipid catabolism;** b** liver expression of the genes related to lipid synthesis *hmgcs1* (3-Hidroxi-3-metilglutaril-CoA -sinthetase 1) *and hmgrc* (3-hydroxy-3-methylglutaryl-CoA reductase). All determined by RT-qPCR and expressed as relative values compared to controls (fold change). ***p < 0.001, **p < 0.01, *p < *0.05 vs. CO;* ^&&^ *p*< *0.01,* ^&^ *p* < *0.05 vs. Hz*

A significant decrease in expression of genes involved in lipid catabolism, such as *acaa 1b* (acetyl-CoA acyltransferase 1), *acat 1* (acetyl CoA acetyltransferase) was found (Fig. 3a). Hepatic expression of the *acaa 1b* gen was normalized in animals deficient in IGF-1 when receiving replacement therapy for 10 days. No significant differences between CO and Hz + IGF-1 were found in *acat1* expression.

**Fig. 4:**
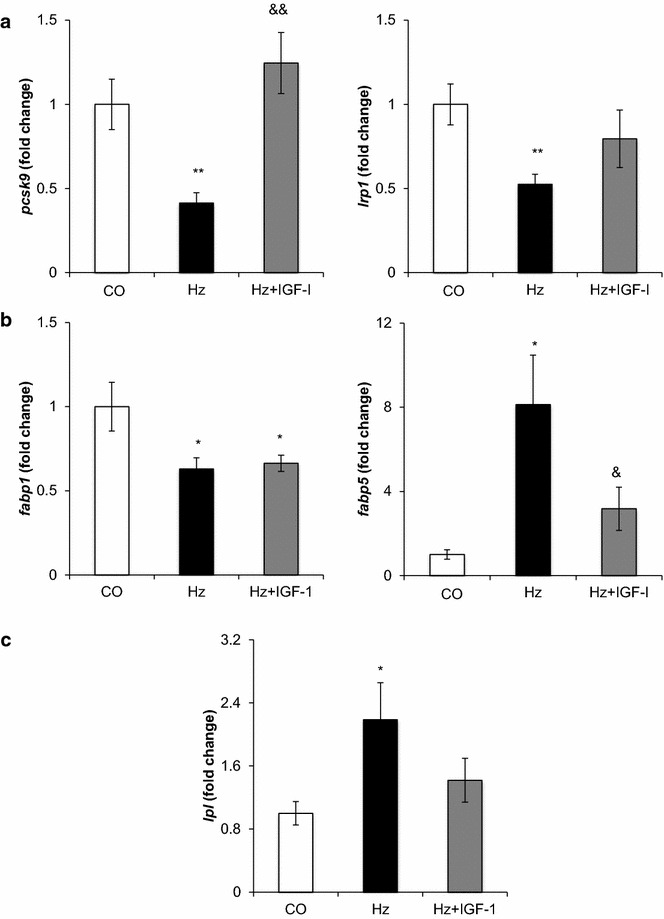
Liver genes implicated in lipid transport.** a** Genes related to low density lipoprotein receptor-related protein synthesis: *pcsk9* (Proprotein convertase subtilisin/kexin type 9) and *lrp1* (Low density lipoprotein receptor-related protein 1);** b** expression of the genes related to fatty acid transport *fabp1* (Fatty acid binding protein 1) and *fabp5* (Fatty acid binding protein 5); and** c** the gene related to triacylglycerol catabolism *lpl* (Lipoprotein lipase). All determined by RT-qPCR and expressed as relative values compared to controls (fold change). **p < 0.01, *p < *0.05 vs. CO;* ^&&^ *p <0.01*, ^&^ *p < 0.05 vs. Hz*

Likewise, partial deficiency of IGF-1 was associated with liver hypoexpression of genes that code for enzymes involved in cholesterol synthesis: *hmgcs1* (3-hydroxy-3-methylglutaryl-CoA synthase1), *hmgcr* (3-hydroxy-3-methylglutaryl-CoA reductase): see Fig. 3b; and those encoding low-density lipoprotein receptor related proteins, *pcsk9* (proprotein convertase subtilisin/Kesin type 9) and *lpr1* (low density lipoprotein receptor-related protein 1). The expressions of all these genes were normalized by IGF-1 replacement therapy (Figs. 3b, 4a).

### Fatty acid transport and triglyceride catabolism

It was found that the partial IGF-1 deficiency resulted in the genetic downregulation of *fabp1* (fatty acid binding protein 1, liver) and an overexpression of *fabp5* (fatty acid binding protein 5, epidermal). RT-qPCR confirmed these findings and showed that *fabp5* was sensitive to replacement therapy with IGF-1 (Fig. 4b).

Furthermore, animals with partial IGF-1 deficiency showed an overexpression of the lipoprotein lipase gene (*lpl*) confirmed by RT-qPCR (Fig. 4c). No differences in the expression of genes encoding proteins involved in the regulation of fatty acid biosynthesis were found (Table 4).

**Table 4:** Liver expression of genes related to lipid metabolism

Protein	Gene	Hz vs. WT (fold change)	p value	Hz + IGF vs. Hz (fold change)	p value
Acetyl-Coenzyme A acyltransferase 1B	*(Acaa1b)*	−4.04	0.002	2.28	0.00012
Acetyl-Coenzyme A acetyltransferase 1	*(Acat1)*	−1.62	0.00006	1.21	0.017
Acetyl-Coenzyme A acetyltransferase 2	*(Acat2)*	−1.50	0.04	1.34	0.02
Acyl-Coenzyme A oxidase 1, palmitoyl	*(Acox1)*	−1.70	0.004	1.14	0.13
Acyl-CoA synthetase long-chain family member 1	*(Acsl1)*	−2.20	0.0001	1.85	0.011
Acyl-CoA synthetase long-chain family member 3	*(Acsl3)*	−1.96	0.018	−1.06	0.19
Acyl-CoA synthetase medium-chain family m2	*(Acsm2)*	−1.09	0.57	1.50	0.002
Acyl-CoA thioesterase 9	*(Acot9)*	1.77	0.008	−1.70	0.006
Cytochrome b5 reductase 3	*(Cyb5r3)*	−1.52	0.04	1.46	0.03
Phenylalkylamine Ca2+ antagonist (emopamil) binding protein	*(Ebp)*	1.49	0.13	−1.16	0.21
3-hydroxy-3-methylglutaryl-Coenzyme A reductase	*(Hmgcr)*	−2.77	0.0018	1.37	0.003
3-hydroxy-3-methylglutaryl-Coenzyme A synthase1	*(Hmgcs1)*	−1.49	0.019	1.45	0.04
Isopentenyl-diphosphate delta isomerase	*(Idi1)*	−1.49	0.013	1.38	0.02
Mevalonate (diphospho) decarboxylase	*(Mvd)*	−2.13	0.002	1.69	0.01
ATP-binding cassette, sub-family G (WHITE), member 1	*(Abcg1)*	1.83	0.004	−1.66	0.05
Aldo–keto reductase family 1, member D1	*(Akr1d1)*	−2.36	0.0002	1.21	0.018
Cytochrome P450, family 46, subfamily a, polypeptide 1	*(Cyp46a1)*	−1.77	0.004	1.01	0.17
Cytochrome P450, family 7, subfamily a, polypeptide 1	*(Cyp7a1)*	−2.76	0.0002	1.54	0.0013
Very low density lipoprotein receptor	*(Vldlr)*	−3.12	0.0002	1.20	0.02
Low density lipoprotein receptor-related protein 1	*(Lrp1)*	−1.49	0.0004	1.13	0.58
Proprotein convertase subtilisin/kexin type 9	*(Pcsk9)*	−1.59	0.03	1.45	0.03
Fatty acid binding protein 1, liver	*(Fabp1)*	−1.53	0.03	1.50	0.007
Fatty acid binding protein 5, epidermal	*(Fabp5)*	5.06	0.0002	−2.27	0.006
Lipoprotein lipase	*(Lpl)*	2.02	0.0012	−2.50	0.002

Control group, WT mice; Hz group including untreated mice with partial IGF-1 deficiency; Hz + IGF-1 group: Hz mice treated with low doses of IGF-1 during 10 days. Underlined values correspond to those with a fold-change >1.5, considered as a significant variation in the gene expression

## Discussion

In the last decades many authors have tried to elucidate the involvement of different factors in the establishment of MetS including impaired glucose and lipid metabolism, lipotoxicity, steatosis, oxidative stress, obesity, interrupted neuro-hormonal regulation and altered intracellular Ca^2+^ trafficking [10–14].

Lately, increasing evidence points to a central role of IGF-1 in the pathological pathways leading to the establishment of MetS [32]. Several recent studies have attempted to correlate low IGF-1 serum levels with MetS [33, 34]. In this context, the present study was focused on determining how the IGF-1 deficiency “*per se*” could contribute to the establishment of MetS.

With this objective, it was used a recently characterized experimental model of haploinsufficiency in which mice hypoexpressing the *igf*1 gene (*igf*^+/−^, Hz = heterozygous) show low circulating IGF-1 levels [26, 31].

The IGF-1 deficiency correlated with a significant reduction in body weight [26] and the replacement therapy was able to restore both IGF-1 serum levels and body weight in accordance with previous results [31].

Data in this paper show that the mere IGF-1 deficiency in adult mice is responsible for altering the hepatic expression of genes involved in glucose and lipid metabolism leading to hypertriglyceridemia, hypercholesterolemia and hyperglycemia (Table 1). The IGF-1 therapy was able to revert all these parameters with only 10 days of treatment.

The adult mice used in this work lived within very controlled conditions (diet, drink, temperature) avoiding all exogenous insults. Consequently, all of the findings in this study are due to the mere IGF-1 deficiency.

One of the most noticeable results in this paper is that the mere IGF-1 deficiency reduced the liver expression of genes coding for phosphoenolpyruvate carboxykinase (PEPCK) and glucose-6-phosphatase (G6Pase), key enzymes involved in gluconeogenesis and both restored to normal values by IGF-1 therapy.

The net glucose release is the result of two simultaneous ongoing pathways that are tightly regulated. The liver produces glucose by breaking down glycogen (glycogenolysis) and by “*de novo*” synthesis of glucose (gluconeogenesis) from non-carbohydrate precursors such as lactate, amino acids and glycerol [35].

The rate of gluconeogenesis is mainly controlled by the activities of unidirectional enzymes such as PEPCK and G6Pase [36]. *Pck1* is involved in the expression of the enzyme that catalyzes one of the rate limiting steps of gluconeogenesis, the conversion of oxaloacetate to phosphoenolpyruvate (PEP), while G6Pase catalyzes the final step of gluconeogenesis, the production of free glucose from glucose-6-phosphate (G6P). Until now, it is well known that the genes of these gluconeogenic enzymes are controlled at the transcriptional level by hormones, mainly insulin, glucagon and glucocorticoids [36].

Interestingly, it is well recognized that insulin decreases the expression of *g6pc* and *pck1.* Results in this paper demonstrate that IGF-1 induces the opposite effects since the IGF-1 deficit reduces the expression of *g6pc* and *pck1.* Thus, these activities of IGF-1 are not “insulin like” but rather antagonistic. These findings reinforce the role of IGF-1 in glucose homeostasis, recently suggested by many authors [5, 37–40].

It is well known that insulin and IGF-1 share structural homology and interact with the same membrane receptors, albeit with different affinities. Insulin and IGF-1 exert complementary biological actions, which are pathologically important when one of the hormone levels drops significantly [14].

In this sense, a different result within this paper, that deserves an especial mention, is the hypoexpression of *pdk4* in IGF-1 deficient mice, which IGF-1 replacement therapy was able to revert. This gene (*pdk4*) encodes pyruvate-dehydrogenase complex (PDK). PDK is an emerging target for the treatment of MetS. To maintain a steady-state concentration of adenosine triphosphate during the feed-fast cycle, cells require efficient utilization of fatty acid and glucose, which is controlled by PDK [41]. Particularly the *pdk4* gene encodes PDK that converts pyruvate, CoA and oxidized nicotinamide adenine dinucleotide (NAD^+^) into acetyl-CoA, the reduced form of nicotinamide adenine dinucleotide (NADH) and carbon dioxide. The activity of PDK is up- and downregulated by pyruvate dehydrogenase kinase and pyruvate dehydrogenase phosphatase, respectively. In addition, pyruvate is a key intermediate of glucose oxidation and an important precursor for the synthesis of glucose, glycerol, fatty acids and nonessential amino acids [41].

As aforementioned, untreated Hz mice 28 ± 6 weeks old showed hyperglycemia as compared to controls and Hz + IGF-1. Interestingly a similar finding was reported in aging rats, which showed low circulating levels of IGF-1 [7]. Unfortunately, in the present work insulin measurements and HOMA assessments are absent due to the little volume of blood that can be extracted from mice.

Results in this paper are in accordance with observations from other authors. It has been recently reported that a postprandial hyperglycemia in patients with Laron Syndrome was due to chronic IGF-1 deficiency and was reversed by IGF-1 replacement therapy [40].

Laron and Weinberger reported in 2004 two cases of untreated patients with congenital IGF-1 deficiency (Laron Syndrome), who developed T2D when they reached 39 and 41 years of age [42]. In our experience (data not published yet), this clinical development in never-treated patients with Laron Syndrome is quite frequent when other factors (especially diet) converge. Another recent study found that sensitivity to T2D is gender dependent in mice with impaired IGF-1 actions, showing that without high fat diet feeding males tend to develop glucose intolerance with age along with insulin resistance, which occurred in both males and females [43].

In addition, untreated Hz mice exhibit a significant reduction in the hepatic expression of *acly,* which was normalized by IGF-1 replacement therapy. *Acly* encodes ATP-citrate lyase, one of the two cytosolic enzymes that synthesize acetyl-CoA. Because acetyl-CoA is an essential building block for cholesterol and triglycerides, *acly* has been considered a therapeutic target for hyperlipidemias and obesity [44]. In the present work untreated Hz mice with a tightly controlled diet and 28 ± 6 weeks old, showed increased circulating levels both triglycerides and cholesterol (Table 1), which the IGF-1 replacement therapy improved.

Accordingly, results in this paper describe a decreased expression of genes encoding several enzymes involved in lipid metabolism: *acaa 1b* (acetyl-CoA acyltransferase 1B), *acat 1* (acetyl-CoA acetyltransferase 1) (Fig. 3a). *Acaa 1b* encodes an enzyme operative in the beta-oxidation system of the peroxisomes. Acetyl-CoA acyltransferase 1 is involved in the regulation of genes encoding cholesterol biosynthesis enzymes in the liver, suggesting that the peroxisome could be a promising candidate for the correction of cholesterol imbalance in dyslipidemia [45].

These data suggest a relevant function for IGF-1 in β-oxidation and cholesterol synthesis. On the other hand, two key enzymes for cholesterol biosynthesis were found reduced in the untreated Hz group and reverted by IGF-1 therapy: hmgcr (3-hydroxy-3-methylglutaryl-Co-A reductase—HMG-CoA reductase), and hmgcs1 (3-hydroxy-3-methylglutaryl-CoA synthase 1—HMG-CoA synthase 1) (Fig. 3b).

The reaction catalyzed by HMG-CoA reductase is rate-limiting for cholesterol synthesis. This enzyme is highly regulated and is a target for pharmaceutical intervention to control hypercholesterolemia [46]. HMG-CoA reductase catalyzes the production of mevalonate from HMG-CoA. The carboxyl group of hydroxymethylglutarate linked by an ester bond to the thiol of Co-A is first reduced to an aldehyde and then to an alcohol. NADPH serves as a reductant in the 2-step reaction [47]. Mevaldehyde is thought to be an active site intermediate, following the first reduction and the release of CoA [47–49].

The enzyme HMG-CoA synthase catalyzes the condensation of an acetoacetyl-CoA and an acetyl-CoA to form HMG-CoA plus free CoA. HMG-CoA synthase activity is found both in the cytosol and in the mitochondria. The HMG-CoA produced by the cytosolic HMG-CoA synthase is converted to mevalonate by the action of HMG-CoA reductase. This reaction starts the isoprenoid pathway, whose main end-product is cholesterol [50].

Although results in this study show a diminished capability for IGF-1 deficient mice in cholesterol synthesis, as *hmgcr*, *hmgcs1* and *acly* (enzymes involved in cholesterol synthesis) are downregulated, augmented serum cholesterol levels were found. This fact could be explained by the downregulation of *lrp1* (LDL-receptor related protein 1) and *pcsk9* (proprotein convertase subtilisin/Kesin type 9) both LDL-receptor related proteins, which could be impairing reverse cholesterol transport from the diet.

Moreover, results regarding lipid metabolism are in accordance with previous studies from this group undertaken in murine aging model, which sustain that IGF-1 and cholesterol have an inverse correlation [7] and are also associated with a mitochondrial dysfunction [51], reversible with IGF-1 treatment. Contrarily, studies undertaken in adult humans [52] disagree with such finding, suggesting that IGF-1 does not have an important role in cholesterol metabolism, but, however, agreed when they found that IGF-1 levels and triglycerides were inversely correlated [52].

On the other hand, a study undertaken in adult individuals showed that IGF-1 levels were positively related with HDL concentrations [52]. Also, in animal models it has been observed that IGF-1 gene expression is higher in adipose tissue compared to other organs [53]. This is in accordance with the fact that IGF-1 could have a lipolytic paracrine effect on adipocytes [54].

IGF-1 circulating levels decline with age. Previously it has been reported that aging rats showed low IGF-1 circulating levels associated to hyperlipidemia (cholesterol and triglycerides), hyperglycemia with insulin resistance [7], as well as an increase of peroxidative liver damage and mitochondrial dysfunction [51]. The exogenous administration of IGF-1, at low doses (similar to those used in the present work), restored IGF-1 serum levels reducing dyslipidemia and insulin resistance, oxidative liver damage and mitochondrial dysfunction [7, 51].

Likewise results in this paper show an increase in MDA homogenate levels, indicative of oxidative damage in the liver of untreated mice with partial IGF-1 deficiency as compared to controls. In addition, IGF-1 therapy induced a reduction of MDA levels even under control values, suggesting an antioxidant activity of IGF-1 according to previously reported results [8].

One recent large-scale community based Framingham Heart Study suggested that lower IGF-1 levels are associated with insulin resistance and MetS [55]. All of these data are in accordance with the observations of reduced IGF-1 levels in individuals with MetS and its various components [14].

The primary condition of IGF-1 deficiency in humans is Laron Syndrome, characterized by low body weight and stature, similar to the one found in mice. However, more recently, IGF-1 deficiency has been associated with an increased prevalence of obesity. A general finding is that obese patients that fulfill the criteria for MetS presenting low IGF-1 plasma levels, tend to develop a worse cardiovascular disease outcome than those with mid-normal to high-normal IGF-1 level [32]. Findings in this work support this idea as metabolic deregulation has been suggested. Although mice with partial IGF-1 deficiency exhibit retarded body weight gain, they have not been exposed to a high fat diet or any other external insult, as they were kept within strictly controlled conditions, thus being IGF-1 deficiency the only feasible component causing metabolic imbalance. Such deregulation found could render these animals to the deleterious effects of obesity if fed with a high fat diet, presumably leading to metabolic syndrome.

In fact, circulating IGF-1 levels are reported to be inversely correlated with the risk of cardiovascular diseases [14]. For example, elderly patients with low circulating IGF-1 levels are at a much higher risk of ischemic stroke and congestive heart failure [56].

Visceral adipose tissue is inversely correlated with circulating IGF-1 levels [33]. In this sense, it was also reported that obesity (always visceral obesity) found in patients with Laron Syndrome is not due to either excessive nutritional intake nor hypometabolism [57].

## Conclusions

In conclusion, our present data, supported by literature evidence, indicates that IGF-1 deficiency is deeply involved in the establishment of MetS while not a definitive factor in its development. Findings in this paper offer a plausible explanation for some of the mechanisms linking IGF-1 deficiency to the establishment of MetS and suggest this condition to be a novel candidate for IGF-1 replacement therapy.

## Abbreviations

CoA: coenzyme A
ADP: adenosine diphosphate
Akt (PKB): protein kinase B
CO: control
DM: diabetes mellitus
FFA: free fatty acids
G6Pase: glucose-6-phosphatase
G6PD: glucose 6 phosphate dehydrogenase
HDL: high density lipoprotein
Hz: heterozygotes
IGF-1: insulin-like growth factor-1
IGF-1R: IGF-1 receptor
IGFBP: insulin-like growth factor binding protein
LDL: low density lipoprotein
MDA: malondialdehyde
MetS: metabolic syndrome
T2D: type 2 diabetes
PCR: polymerase chain reaction
PKC: protein kinase C
PI3K: phosphoinositide 3-kinase
IR: insulin receptor
VLDL: very low density lipoprotein
WT: wild type
